# Understanding young adults’ reasons for seeking ‘clinically unnecessary’ urgent and emergency care: A qualitative interview study

**DOI:** 10.1111/hex.13301

**Published:** 2021-06-12

**Authors:** Jaqui Long, Emma Knowles, Lindsey Bishop‐Edwards, Alicia O’Cathain

**Affiliations:** ^1^ School of Health and Related Research (ScHARR) University of Sheffield Sheffield UK

**Keywords:** emergency medicine, health care–seeking behaviour, health service use, qualitative research, urgent care, young adults

## Abstract

**Background:**

Studies have identified young adults as more likely to use emergency departments for ‘clinically unnecessary’ problems, with limited similar evidence for emergency ambulance use. Media portrayals depict young adults as motivated by ‘convenience’, but little research has explored the reasons for their help‐seeking behaviour.

**Methods:**

Qualitative interviews with 16 young adults (18‐30) considered by clinicians to have made unnecessary use of emergency ambulance, emergency department or an urgent GP appointment. Data analysis was informed by interpretive phenomenological analysis.

**Findings:**

A number of interrelated factors contributed to participants’ decisions. They were anxious about the seriousness of their symptoms, sometimes exacerbated by reduced coping capacity due to poor mental health or life stresses. They looked to others to facilitate their decision making, who sometimes encouraged urgent contact. They wanted to avoid impact on existing day‐to‐day commitments including work or study. They had strong views about different health services, sometimes based on frustration with lack of resolution of on‐going health problems. Convenience was not identified as a significant factor, although some actions could be interpreted in this light if the context was not considered.

**Conclusions:**

Young adults make ‘clinically unnecessary’ use of urgent and emergency care for more than convenience. Their decisions need to be understood in relation to the complexity of their experience, including lack of confidence in making health‐related decisions, lowered coping capacity and concern to maintain normal daily life.

## 1 BACKGROUND

Urgent and emergency care services in many countries are under increasing pressure due to growing demand that has not been matched by an equivalent expansion in resources.^1^, ^2^ In the UK, challenges are particularly acute for the emergency ambulance service, emergency departments (EDs) and urgent same‐day general practice (GP) appointments.^3^, ^4^, ^5^, ^6^ A particular concern is that some demand may be due to patients using services for problems that do not need such a high‐acuity service or could be managed through self‐care.^7^, ^8^ Such demand is described as using services for non‐urgent, medically unnecessary or low acuity problems, or as ‘inappropriate use’.^9^, ^10^, ^11^, ^12^ In this article, we use the term ‘clinically unnecessary’, recognizing that these definitions or judgements are from clinicians’ perspective, and that patients’ decisions may be driven by other, non‐clinical, considerations.

Studies have sought to identify whether specific groups are disproportionately likely to make clinically unnecessary use of urgent and emergency care and to explore the reasons for this. Young adults have been identified as one such group in relation to EDs,^11^, ^12^, ^13^ with limited evidence of a similar pattern for emergency ambulance.^14^, ^15^ However, few studies have explored why this might be the case. There is a widespread belief that young adults epitomize a growing trend towards convenience‐driven use of urgent and emergency care, in particular by showing a preference for EDs rather than general practice.^16^, ^17^, ^18^ Media portrayals often depict young adults as being used to a 24/7 culture and expecting services to be immediately available, or using EDs due to their chaotic lifestyles.^19^, ^20^ In research, a recent study exploring use of a range of urgent care services (111, ED, minor injuries unit, urgent care centre) found young adults identified the lack of alternatives as their reason for using EDs.^21^, ^22^ This could be driven by lack of access to GP services,^20^, ^23^ lack of knowledge of alternatives or confusion about how to use them.^21^, ^22^ Overall, there is limited understanding of why young adults use urgent and emergency care services for clinically unnecessary problems.

**FIGURE 1:**
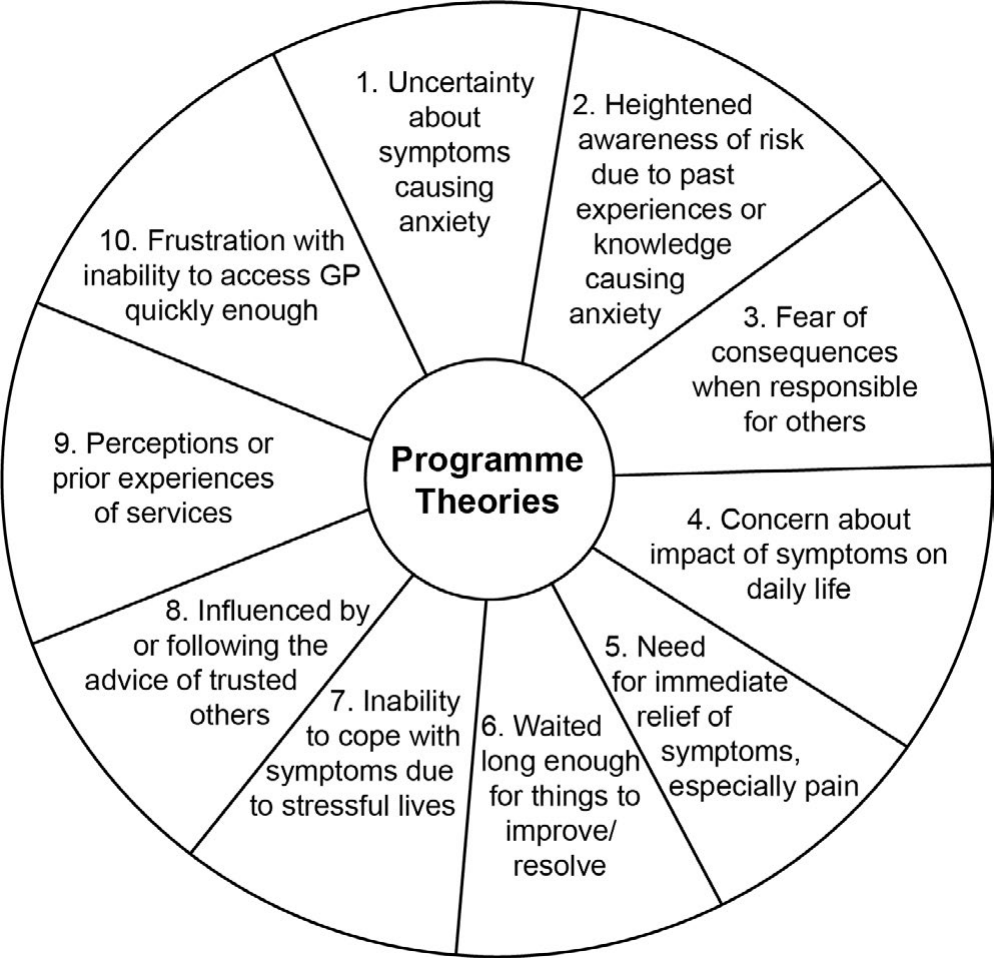
Programmes theories developed from realist review

In 2018/19, we conducted a mixed‐methods study to understand clinically unnecessary demand for the ambulance service, EDs and urgent GP appointments.^24^ We focused some of this research on three groups perceived to be more likely to be clinically unnecessary users: young adults, parents of young children and those living in areas of deprivation. The study included a realist review of qualitative literature,^25^ through which we developed ten ‘programme theories’ (PTs) to explain help‐seeking decisions (Figure 1). We did not identify ‘convenience’ as one of our PTs, although it was possible to see how aspects of people's decisions could be interpreted as such, for example wanting to reduce the impact of symptoms on daily life, seeking immediate pain relief or inability to access GP quickly enough. We tested the PTs through exploring theoretical literature relating to health behaviour and found Wyke et al's^26^ work reflected the complex process we had identified. Synthesizing three existing models of health behaviour,^27^, ^28^, ^29^ their ‘Concentric circles of influence’ model suggests that individual's interpretation and response to their symptoms are developed as a result ‘of accumulated knowledge and through interactions’^26^ (p79). A range of factors influences this interpretation, including the degree to which symptoms impact on normality, how they are understood (including expectations of outcome) and the personal and wider resources available to an individual. Additionally, individuals are embedded within and fundamentally affected by their social and cultural networks. Changes in any one aspect of this complex system, including physical or emotional state, knowledge or contact with others, may influence how symptoms are interpreted and action taken.

While young adults were included in the study samples in our realist review, none of the literature specifically focused on them. To gain greater insight, we conducted qualitative interviews to explore their perspectives and experiences. In this paper, we present and discuss the findings relating to young adults’ decision making when seeking ‘clinically unnecessary’ care from the emergency ambulance service, ED or an urgent GP appointment.

## 2 METHODS

### 2.1 Ethical approval

Approval was obtained from London Brent Research Ethics Committee (ref 14/LO/1228).

### 2.2 Patient and public involvement (PPI)

The study was initially discussed with Sheffield Emergency Care Forum, a PPI group specializing in emergency care research. PPI co‐applicants attended project management and advisory group meetings throughout the study. The interview topic guide was piloted with two PPI co‐applicants, which led to some revisions. PPI co‐applicants also contributed to data analysis by discussing the initial themes identified and their interpretation.

### 2.3 Setting and participants

Two cities with different service configurations were selected, one in the Midlands and one in the North of England. For our study, we initially defined the age range for young adults as 18‐24, but due to recruitment difficulties, this was extended to 18‐30.

### 2.4 Recruitment

In our wider study, we aimed to recruit 16 participants to each group (young adults, parents, deprivation) with purposive sampling from different services and maximum diversity of socio‐demographic characteristics. We recruited from seven service locations: two ambulance services, three EDs including one specialist children's ED and three GP services. In each service, clinicians identified patients they considered had made ‘clinically unnecessary’ use of the service; that is, the patient could have used a lower acuity service or self‐care. In the ambulance service and GP practices, staff within the service obtained patients’ permission to securely share their details with the research team for follow‐up. In the EDs, a member of the research team attended the department and provided initial information directly to relevant patients identified by clinicians. To avoid conveying a sense of criticism, the study was explained in terms of understanding people's decision making regarding use of different health services; patients were not informed of the clinician's judgement.

Despite clinicians’ enthusiasm for the study, we experienced significant recruitment challenges to the study. Clinicians did not always remember to speak to potential patients and found it unexpectedly difficult to confidently identify ‘clinically unnecessary’ users. Despite multiple attempts, we were unable to contact nearly 30% of those initially identified, and more than 10% declined when contacted; these challenges were particularly acute in relation to young adults, together with a lower level of initial interest from those approached in the EDs. While we intended to recruit as diverse a sample as possible, time limitations meant we interviewed any participants recruited and stopped recruitment when we reached our target of 16.

### 2.5 Data collection

Once informed consent was obtained, the interview followed a broad topic guide (Supplementary File 1) to ensure key areas were covered. Questions were not based on the PTs, but instead explored the events leading up to their contact with the particular service, including any advice sought or steps taken before the contact; and experiences, awareness and perceptions of different services. At the end of the interview, demographic data were collected and participants were given a shopping voucher to thank them for their time. Interviews were conducted by one of two experienced qualitative researchers at the university (9), in participants’ homes (6) or by telephone (1), within two weeks of the service contact where possible. Interviews lasted 22‐52 minutes (average 37 minutes) and were audio‐recorded for later transcription by the university's service.

### 2.6 Data analysis

Four members of the research team were involved in the analysis (EK, JL, LBE and AOC) and met regularly throughout. The principles of interpretive phenomenological analysis (IPA) were used to analyse the data. IPA aims to explore individuals’ lived experience in a particular context and focuses on their perceptions of events and how they make sense of their experience.^30^ Each case is analysed as a whole, using a predominantly inductive approach, which allows the essence of the experience to be identified.^30^, ^31^ While IPA is generally used with small sample sizes, it offers guidelines that can be adapted for larger samples.^30^, ^32^

**FIGURE 2:**
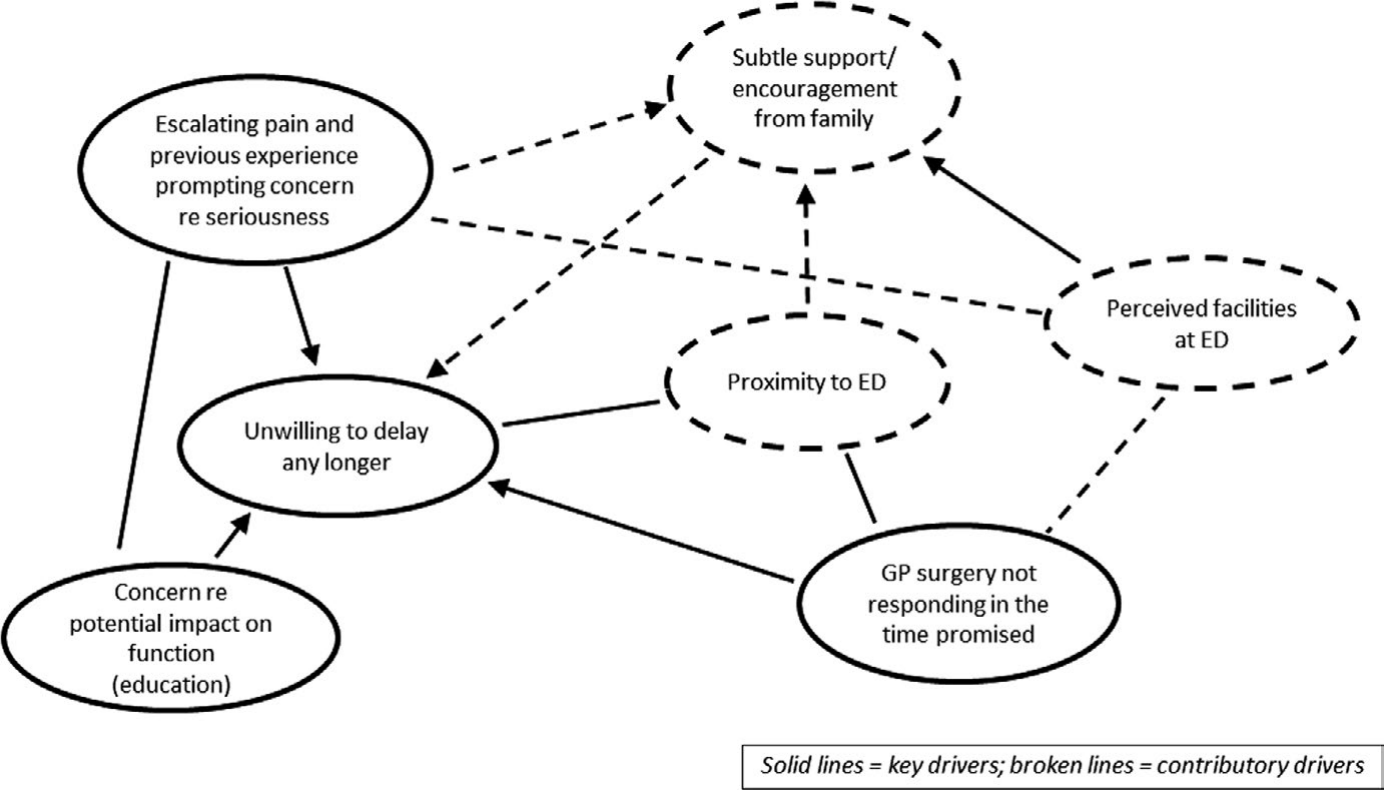
Diagram of drivers for participant P2

For each interview, one researcher reviewed the transcript and created a memo and diagram (see Figure 2 for an example) identifying and representing the drivers for the participant's decision making. Where possible, drivers were identified in terms of whether they were seen as key to the decision or contributory to it. Each memo and diagram was discussed and agreed with a second team member, with the interviewer always being part of this process. The PTs were not used to structure the analysis but once the memos and diagrams were finalized, one member of the team who had also been involved in the review identified the correspondences between the drivers and the PTs, which were then agreed by the team (see Table 2).

## 3 RESULTS

### 3.1 Participant characteristics

Participants’ mean age was 25 (range 18‐30); only two teenage participants were recruited. Most were White British, female and in full‐time employment or study. Three were unemployed, predominantly due to health reasons. Half the sample were living in areas of social deprivation, and four were parents. Full demographic details are in Table 1. Most participants had sought help from the ED (8) or GP (6), with only two using the emergency ambulance service. Almost all participants living in areas of deprivation had contacted the ED or emergency ambulance service, compared to less than half of those in more affluent areas.

**TABLE 1:** Participants’ socio‐demographic details

Gender	
Male	5
Female	11
Age	
<20	2
20‐25	6
26‐30	8
Length of residence in city	
<1 y	1
1‐5 y	4
>5 y	11
Ethnicity	
White—British	14
Asian/Asian British—Pakistani	1
Asian/Asian British—Chinese	1
Marital status	
Married/living as married	3
Single/not married	13
Employment status^a^	
Working full time	5
Working part time	4
Student (full time)	6
Temporarily unemployed, seeking work	2
Permanently unemployed (illness/independent means)	1
Other—unemployed/unemployed due to bad health	1
Other—self‐employed and part‐time study	1
Parent	
Yes, child under 5^b^	2
Yes, child over 5^b^	3
No	12
Living in area of deprivation (IMD 1‐3)	
Yes	8
No	8

^a^ This section adds up to more than 16 due to a number of people selecting more than one option.

^b^ Four participants were parents, one had two children, one under 5 and one over 5.

Interviewees reported seeking help for a wide range of reasons: exacerbations of chronic or on‐going physical symptoms (5) including pain, bleeding, digestive problems and fatigue; pain after injuries (4); seeking medication to manage anticipated rather than actual symptoms (2); severe anxiety (1); breathing difficulties after chest infection (1); suspected concussion (1); vomiting blood after drinking alcohol (1); and suspected miscarriage (1).

Participants frequently reported a complex picture, with all describing a number of drivers for their decision. Interviewees identified between two and seven drivers (average 4‐5) which were usually interconnected, as illustrated in Figure 2. Six drivers featured most strongly, which are detailed in Table 2 together with their relationships to the PTs.

**TABLE 2:** Relationship between themes from interviews and programme theories from review

Themes from the qualitative interviews	Correspondences identified with programme theories (PTs) from realist review	
	Key PT	Additional PTs also present
Concern about seriousness of symptoms and desire for reassurance	1. Uncertainty about symptoms causing anxiety	2. Heightened awareness of risk due to past experiences or knowledge, causing anxiety
Reduced coping capacity due to poor mental health, stress, lack of resources	7. Inability to cope with symptoms due to stressful lives	1. Uncertainty about symptoms causing anxiety 5. Need for immediate relief of symptoms, especially pain
Influence of others, particularly social networks	8. Influence of others	1. Uncertainty about symptoms causing anxiety (when others not present) 9. Perceptions or prior experiences of services (other people's)
Concern about the impact of symptoms on daily life	4. Concern about impact of symptoms on daily life	10. Frustration with inability to access GP quickly enough
Positive and negative views of different services	9. Perceptions or prior experiences of services	10. Frustration with inability to access GP quickly enough
Frustration at lack of resolution of an on‐going problem, despite previous efforts	6. Waited long enough for things to improve/resolve	9. Perceptions or prior experiences of services 10. Frustration with inability to access GP quickly enough

### 3.2 Concern about the seriousness of symptoms and desire for reassurance

Most participants described seeking health care because they were concerned their symptoms could indicate a serious health problem, and did not want to delay getting assessment or treatment. Uncertainty about the meaning of symptoms was a frequent cause of anxiety, and unfamiliarity was a particularly significant trigger, in relation to both psychological and physical symptoms. This was sometimes exacerbated by online information‐seeking.

I just didn't know what was wrong with me at the time it was just really scary… I was just frightened it had just not sort of happened before… Just a really heightened sense of fear and the whole sense of doom thing was happening and ‐at the time ‐ it felt very urgent. Yeah I'd, I'd never had it like that before so it’s just totally new [P10 GP]

…I mean like the daft decision is searching the symptoms online, and it just freaked me out […] all sorts of like, I don’t know, just big stomach issues, something like ulcers or obviously cancer is always on it. [P3, ED]

While unfamiliarity was a frequent driver, for a smaller number of participants experiencing recognizable symptoms could also increase the sense of urgency. For some, it suggested the return of a previous health problem, while others recalled the serious consequences similar symptoms had had for someone else. The severity of symptoms could be another key trigger, mostly in relation to injury rather than illness. Sometimes a progressive worsening despite self‐care measures introduced an element of unfamiliarity. On other occasions, the severity was immediately apparent and sufficient in itself to trigger action:

As I fell off I landed awkwardly on my right ankle and just completely rolled it. And then at that point it felt – it kind of just went straight away it went really hard – and as dramatic as it sounds I thought there was a bone sticking out. I couldn’t dare look because it just hurt [P16, ED]

Concern about the seriousness of the symptoms was sometimes accompanied by a specific desire for reassurance from a health professional. Some participants described thinking that their problem was unlikely to be serious, but wanting to rule out a nagging concern, for example: ‘to double check that it's not like a break or a twist or like a torn thing’ [P3, ED].

### 3.3 Reduced coping capacity due to poor mental health, stress or lack of resources

The wider context of participants’ lives often appeared to affect their help‐seeking. A third of the sample referred to the influence of mental health problems, including generalized anxiety and depression, on their decision making. In most instances, participants were seeking help for physical concerns, but described a complex interplay between mental and physical health, with their on‐going struggle with their mental health reducing their ability to function and cope with the challenges of additional physical symptoms. In particular, underlying anxiety sometimes intensified participants’ concern about their symptoms, increasing the perceived need for urgent help and reassurance. For one participant, severe anxiety leaving home may have influenced the decision to call an emergency ambulance due to the challenges of using other services.

Other participants described how high levels of stress sometimes triggered an exacerbation of existing physical symptoms to an intolerable level, impacting the urgency of help‐seeking:

…just like work, relationship, stress you know, all that stuff, so I know that that is what has been making it a trillion times worse. (P13, GP)

Finally, financial considerations influenced a few participants’ choice of service. For example, one participant, who lived in an area of deprivation, cited the cost of transport as contributing to their decision to choose the ED over the more distant Walk‐In Centre.

### 3.4 Influence of others, particularly social networks

For most participants, other people had some degree of influence over their decision. Most advice came from family, friends and work colleagues, sometimes based on their own experiences. This particularly related to ED attendance and suggested a social norm in the perception of this service and its role:

… one of my colleagues a couple of months ago had done similar thing and sprained it, I asked him where he went because I didn't right know, I were gonna go to Walk‐in Centre but then he said 'there's no point because they don't do X‐rays I don't think, which you'd need' so that's when I went to [ED] (P14, ED)

The degree of influence ranged from making a decision on the participant's behalf when they considered them unfit to make it, encouraging a specific course of action, to shared decision making or supporting a participant's intended action. Conversely, the absence of others could also impact decision making, such as when a participant's problem occurred late at night, making it difficult to draw on their social network for support and reassurance:

I honestly thought I was going to die. I really, really thought I was going to die because I’d never felt like that in my life. Like I said, being on my own as well, nobody to speak to, nobody to reassure me. It was just so scary. (P4, Emergency ambulance)

In two instances, participants were specifically advised to attend the ED by health‐care professionals, one during a consultation with their GP and the other by NHS 111 after being unable to speak to their GP:

(…) the 111 lady spoke to a nurse that – to get a bit of advice about whether I could go to the Walk‐in Centre, she said you wouldn’t be able to go to the Walk‐in Centre but actually we would say go straight to the hospital for stuff like that. (P15, ED)

### 3.5 Concern about the impact of symptoms on daily life

Nearly half the participants expressed unwillingness to delay help seeking to avoid the impact, or potential impact, of symptoms on their ability to function, including work, study, childcare and leisure activities:

I’ve been feeling under the weather generally for a little while but that particular week I’d just had a really difficult week, I couldn’t, I was so tired and fatigued and I couldn’t really eat, do my job properly, so I was struggling with driving and just being at work so I wanted to try and get it looked at. (P9, GP)

…I’d had a headache and I woke up and it was even worse, so I just rang them [GP] up and just said you know I can’t deal with this now. I’ve got a toddler to look after, is there anything that I can do now. (P6, GP)

The need to fulfil significant commitments and ‘get back to normal’ often combined with other factors in influencing participants’ decision making. In particular, for some who sought care from the GP, an inability to obtain a routine appointment within an acceptable time or that fitted with inflexible work responsibilities was a major contributory factor to seeking a same‐day appointment. One participant specifically noted how unpredictable work commitments made it impossible to book a routine appointment some weeks’ ahead, which was often the only alternative to a same‐day appointment. In two cases, no symptoms were present at the time of contact, but participants’ familiarity with their existing health conditions prompted a decision to obtain urgent help. One participant reported running out of an essential medication and being unexpectedly unable to access it through the usual automated route, while the other needed a new prescription to manage potentially troublesome and distressing symptoms during an upcoming holiday. In this second instance, the situation was complicated by the participant having a developmental disorder which meant that disruption to planned activities significantly affected their well‐being. This illustrates the complexity of factors that may not be immediately apparent, but can considerably affect someone's coping capacity and consequent decisions.

### 3.6 Positive and negative views of different services

Most participants’ decisions, particularly those living in areas of deprivation, were influenced by their perceptions and experiences of different services. In relation to the emergency ambulance service, both participants expressed the need for a rapid response they believed only this service could offer. One described her confidence in their speed and skills to assess and deal with her problem, a perception partly driven by previous positive experiences of the service, including their reassurance that she had ‘done the right thing’ (P4) to call. Another, without access to their own transport, described the service as offering safe, easy and rapid transport to hospital.

The majority of participants who sought care from an ED did so either due to a belief that it offered the best or most appropriate service, or conversely that other services could not meet their needs. Beneficial characteristics identified were the speed of access to help, the range of resources and facilities, and the ability to deal with serious problems. Proximity to home or workplace was a contributory rather than a key driver for a few participants. Negative aspects of other services were also sometimes significant. Delays or difficulties in accessing a GP were a particular source of concern, with a few participants describing being unable to get an appointment or speak to someone quickly enough. Occasionally, specific difficulties in participants’ relationship with their GP also contributed to a reluctance to contact this service.

All participants who had successfully contacted a GP expressed a positive perception of this service as being the most appropriate to their needs. GPs were variously seen as a trusted source of knowledge; providing an opportunity to discuss concerns about on‐going or recurrent health problems or obtain a diagnosis; the best place to review medication and obtain repeat prescriptions; and a quick and accessible route to obtaining help:

… if I’m feeling a bit run down at all, or cold, or my chest is bad then they will get me in, on that same day regardless cos obviously it’s a bit more of a medical emergency I guess, more urgent … (P11, GP)

While existing perceptions and experiences were important, in a few instances a lack of awareness of potentially appropriate services also contributed to participants’ decisions. In particular, the minor injuries unit could probably have met the needs of three participants who accessed the ED, but this option was not considered due to lack of knowledge of the service.

### 3.7 Frustration at lack of resolution of an on‐going problem, despite previous efforts

In a number of instances, participants’ concern did not arise out of an isolated incident, but was part of an on‐going process of seeking resolution for a longer‐term health problem, and the decision to seek help generally followed attempts to manage through self‐care. All had had previous contact with the health‐care system in relation to their problem, either through the GP and/or hospital services, but this had not as yet provided a satisfactory outcome. In most cases, an aggravation of their symptoms prompted their urgent contact, but there was an underlying sense of an on‐going struggle to tolerate their situation and its impact:

[I was] really sick of it, really and just having all these symptoms, and for all these tests that I’ve had to come back clear. It’s like well there is something wrong with me […] I need to pursue it, and try and find out. [P13 GP]

Most of these participants sought an urgent appointment with the GP, either being unable to secure a routine appointment quickly enough to meet their needs or because work commitments made these difficult to access. In a few instances, lack of confidence in their GP or lack of response to their initial call within a tolerable timeframe prompted use of the ED, where they felt more confident of receiving rapid and appropriate help.

## 4 DISCUSSION AND IMPLICATIONS

Although convenience has been proposed as a significant factor in young adults’ use of urgent and emergency care for clinically unnecessary problems, in our sample a range of interrelated factors contributed to their decisions. These included feeling anxious about the seriousness of symptoms, sometimes exacerbated by reduced coping capacity due to poor mental health or other stresses; being influenced by others, particularly family and friends; wanting to fulfil daily commitments and maintain ‘normal life’. Previous experiences or perceptions of services, sometimes including frustration with unresolved on‐going health problems, were also a significant influence.

There were clear correspondences between our findings and the PTs identified in the realist review (see Table 2), which suggests that overall the factors affecting young adults are the same as for other age groups. This was supported by data from a population survey of attitudes to help seeking undertaken as another part of our study,^33^ which found that ‘uncertainty’ (PT1), ‘impact on daily life’ (PT4), ‘stressful lives’ (PT7) and ‘perceptions of services’ (PT9) most commonly explained clinically unnecessary service use.^24^ However, both the interview and survey data suggest some distinctive aspects to young adults’ decision making.

Compared to the other two groups of interview participants (parents of young children and those living in areas of deprivation), young adults more frequently reported being influenced by a concern over the impact of symptoms on daily life and functioning, particularly work or study. This is consistent with the desire for equilibrium and ‘normality’ which Wyke et al highlight as a key factor in how individuals interpret and respond to their symptoms.^26^, ^27^ Amiel et al^34^ found similar themes in relation to urgent care centres: younger adults were more likely to use this service and identified proximity to work or home and access to a faster appointment as key reasons for their choice. These findings, combined with perceived difficulties in accessing GP appointments, may provide some insight into the trend identified for this age group to bypass GP services.^20^, ^23^ This has significant implications for primary care scheduling, highlighting the need to explore ways to improve access to ‘work‐friendly’ appointments, while taking account of the pressure this service is already facing. In this context, workplaces have a role in providing more support and flexibility to employees to enable them to access GP appointments during ‘normal’ working hours.

In our sample, young adults living in areas of deprivation more frequently used the higher acuity ED and ambulance services rather than their GP and appeared to be more influenced by their perceptions of services, perhaps suggesting their experiences of these services were a more significant factor in their decision making. Our sample is too small to draw definite conclusions, but these findings are consistent with existing evidence of the link between deprivation and clinically unnecessary use of urgent and emergency care,^16^, ^35^ and of GP provision tending to be poorer in areas of deprivation.^36^ While not unique to young adults, these complex connections need further exploration.

While interview participants across all groups were strongly influenced by concern over the seriousness of symptoms and a desire for reassurance, our survey found those in the 18‐24 age group were likely to feel less confident in deciding when to go to a doctor with a health problem, and to worry that pain indicated a serious problem.^24^ Additionally, those aged 18‐44 were more likely to feel overwhelmed when they had an unexpected health problem.^24^ These data echo Turnbull et al's findings that young adults more frequently identified themselves as anxious and prone to worrying about their health and linked this to their use of urgent and emergency services.^22^ This anxiety and lack of confidence may be an age effect: young adults may be facing unfamiliar symptoms for the first time and be less confident in making independent decisions about their seriousness, and have less familiarity with services. If so, then concerns that this group are introducing a cohort effect that will build as they move through the health‐care system may be unfounded. However, the interview data also suggest other factors may be contributing to this pattern.

Across all interview participants, young adults most frequently reported the impact of stress or poor mental health on their decision making, with a number describing mental health symptoms of varying severity, some seeking treatment through their GP. Even for those reporting less severe difficulties, their help seeking was evidently impacted by their reduced coping capacity. This is consistent with Wyke et al's emphasis on how individuals’ resources and wider life context, including their emotional well‐being, affect their interpretation and action in response to symptoms.^26^ Poorer mental health can heighten anxiety and reduce coping capacity, prompting use of a quick and easy route to health care. Mental health problems or self‐perceived stress, even when not at a clinical level, have been found to be related to increased use of both EDs and primary care.^37^, ^38^, ^39^, ^40^ A significant number of these ED visits are considered ‘avoidable’, with patients being discharged without treatment.^41^ In primary care, users with mental health problems report poorer experiences and more unmet need.^39^ These data suggest that the health‐care system is sometimes failing to meet these patients’ needs, potentially prompting clinically unnecessary service use. Rates of ED use for mental health problems are rising particularly rapidly for adolescents and young adults,^42^, ^43^ and if this age group are being disproportionately affected by these problems, this will inevitably be reflected in their service use. Identifying how mental health provision can better meet young adults’ needs therefore has the potential to benefit both patients and urgent and emergency care services.

Increased anxiety or lack of confidence is liable to prompt people to turn to others for help and advice, and the influence of social networks was clear within the interview data. This was supported by our survey results, where younger adults were significantly more likely to say they would check with family and friends to decide what to do when faced with an unexpected health problem.^24^ Turnbull et al also found young adults were more likely to describe the impact of social context on their help seeking, and for their help‐seeking strategies to be affected if their problem occurred when they were alone or at night, that is when lacking support.^22^ Wyke et al emphasize the importance of social interaction, placing it near the centre of their ‘circles of influence’.^26^ Drawing on Dixon‐Woods’ work around ‘candidacy’, they highlight not only its direct effects in terms of advice or support for an individual's decision but also the subtle ways in which social and cultural norms affect beliefs about symptoms, services and how they should be used.^44^

Our participants also described how health professionals’ advice was sometimes a key factor in their decision making. This is consistent with McKenna et al's findings that people in positions of authority, particularly health‐care workers, were a significant factor in contributing to individuals’ avoidable ED attendance.^45^ The challenges encountered by clinicians recruiting participants for our study highlight the difficulties faced in making appropriate decisions regarding clinical necessity, particularly in a context of time pressure and avoidance of risk.

In contrast to media articles and some other research, we did not find convenience to be a strong influence in participants’ decision making. While this may be a reflection of our sample, it also highlights that some young adults’ decisions are driven by a complex range of factors. When considered individually, some of these factors could be interpreted as convenience, including fitting health care around work commitments; proximity of service to work or home; lack of personal transport; and avoiding disruptive symptoms during a holiday. In all these instances however, when the complexity of the participant's situation was taken into account, the decision could be understood in a different light.

## 5 STRENGTHS AND LIMITATIONS

This is one of the first studies to explore young adults’ perspectives in relation to clinically unnecessary use of urgent and emergency care services. However, this is a self‐selected group of participants, and the sample was difficult to recruit and therefore may not be representative of the wider age cohort. It is perhaps likely that patients using services for ‘convenience’ were less willing to participate in the study, either for fear of criticism or because taking part in research would also be perceived as inconvenient. There is a need to explore the applicability of our findings to a more diverse group of young adults, particularly those in the youngest part of the age band and young adults from Black and Minority Ethnic communities. A larger sample would allow exploration of the diversity of life stages among young adults—for example student, employed, parent—and how these may impact on decision making.

## 6 CONCLUSION

Young adults make clinically unnecessary use of urgent and emergency care for a complex range of reasons. While some of this can be interpreted as convenience, it is important to take into account how limited resources, whether personally or within the health system, can impact on decision making. This includes the broader social context, heightened anxiety and lack of confidence, the influence of others and a need to maintain daily activities and commitments. To fully understand young adults’ decision making, it is important to explore the complexity beneath the ‘convenience’ label.

## CONFLICT OF INTEREST

All authors declare that they have no conflicts of interest.

## Supporting information

Supplementary Material

## Data Availability

Data available on request from the authors.
