# Supplementary material for: Understanding young adults’ reasons for seeking ‘clinically unnecessary’ urgent and emergency care: A qualitative interview study
Source: Health Expect. 2021 Jun 12;24(4):1535–44. doi: 10.1111/hex.13301 (PMC8369113; doi:10.1111/hex.13301)
Supplement: Supplementary file 1 — Supplementary Material [file HEX-24-1535-s001.docx]

**Interview topic guide**

1. Thinking about your recent episode [at service]. Can you say a little about what happened?

| Probe:   - What was the health problem? - How long had you experienced symptoms? - Had you tried any self-care? - Was it a problem experienced before? - Was there anything that particularly worried you about the situation? |
| --- |

2. You made contact with [X service] on [day of week]. Was there a reason why you made contact on that particular day/time? (ie rather than another day that week/another time that day)

| Probe:   - Urgency of health problem - Work commitments, childcare - Transport [if appropriate] - Why at that particular time of day? Had something changed? |
| --- |

3. Prior to contacting the service, had you sought help from anywhere else?

| Probe:   - Any other services you tried to access by phone or in person? Where and when? - What was the outcome? Has that happened before when you’ve tried to get help from this service? |
| --- |

4. Were there any other health services which you *think could have* helped with your problem?

| Probe:   - Did you think about other options? Suggest options if they don’t mention (list other options depending on problem, service contacted and location e.g. NHS 111, walk-in centre, minor injuries unit, out of hours GP etc) - Were you aware of these, why did you choose not to make contact? - What did you think would have been different if you had contacted them? - Have you tried contacting this service in the past? Did this affect your decision? - Did you look online for information/advice? If so, did this affect your decision about what to do? |
| --- |

5. Did anyone else help you decide what to do?

| Probe:   - Who did you talk to about what was happening? Family, friends… - Did they have the same view of your problem (same, more/less serious etc?) - Did anyone else have any different suggestions about what you did? - If you disagreed with them, how did you decide what to do? |
| --- |

6. Thinking more generally, I’d like to ask you a bit more about how you use healthcare services. Generally when you are ill, what is your usual source of healthcare?

| Probe:   - What are the reasons why this is your usual source? Convenience, confidence - How do you decide where you go to get help? - What other services have you used? |
| --- |

7. What did you think would be best about x service for you on this occasion compared to other options/services?

| Probe:   - Anything particular they could offer or anything that the others don’t offer? - Have you ever used this service before? Did this make a difference to your decision? |
| --- |

8. We’ve been talking today about emergency and urgent care services, and we’re interested in finding out what people think these words mean.

| Probe:   - What do you think is the difference between emergency and urgent care? - What things about your situation made you decide it was urgent/an emergency? |
| --- |

9. Are there any changes that could be made to the current health system to make it easier for you to access healthcare?

| Probe: availability of services and information. |
| --- |
